# A Review on the Machinability Enhancement of Metal Matrix Composites by Modern Machining Processes

**DOI:** 10.3390/mi15080947

**Published:** 2024-07-24

**Authors:** Pallab Sarmah, Kapil Gupta

**Affiliations:** Department of Mechanical and Industrial Engineering Technology, University of Johannesburg, Doornfontein Campus, Johannesburg 2028, South Africa; psarmah@uj.ac.za

**Keywords:** metal matrix composites, modern machining, EDM, laser, abrasive, surface quality, productivity

## Abstract

These days, metal matrix composites (MMCs) are being widely utilized in automotive and aerospace industries as prominent alternatives to traditional materials. Owing to their elevated strength-to-weight proportion, exceptional fracture toughness, and lightweight design, they can be used in a variety of applications. MMCs undergo extensive machining while making parts and components out of them. The machining of monolithic materials, such as metals and alloys, is a widely used and established process in different industries, such as the aerospace, bio-medical, and automotive sectors. Because of the properties of the metal matrix and the strong reinforcement, MMCs provide unique challenges. Modern machining processes have been found to be superior in overcoming challenges and achieving improved machinability of MMCs. An overview of MMC machining with modern methods is provided in this article. This article first outlines MMCs and addresses the need for and difficulties associated with their machining. Next, it reviews previous investigations on the machining of MMCs employing modern methods like electrical discharge machining, laser machining, abrasive machining, and hybrid machining. Productivity and surface integrity issues, including delamination and roughness, etc., are discussed. When presenting the review, the benefits and drawbacks of modern processes are also taken into account.

## 1. Introduction

### 1.1. Overview of Metal Matrix Composites

With the expansion of technology and rising demand for diversified materials for different applications in recent decades, material science is exploring new horizons. These materials are extensively developed to be lightweight, be highly resistant to corrosion, have greater fatigue resistance, and have excellent temperature resistance [1]. Two or more physically and chemically interconnected phases constitute a composite material. Various types of composite materials can be found in nature, including wood that is made of cellulose fiber reinforced in a lignin matrix. In the early 1950s, composite materials were created using plastic as a matrix and glass as reinforcement. Composite materials have better characteristics than monolithic alloys and have been frequently used in building, aircraft, and defense since the 1960s [2].

Metal matrix composites (MMCs) have been procured for a long time; however, in the latter part of the twentieth century, they became known as accurately engineered composite materials. MMCs have been employed for high-performance aeronautical applications, electronic packaging, automotive, and bio-medical products [3]. Numerous innovations and developments have been made for different applications. These materials have been effectively used in the aerospace and automotive industries, as a substantial amount of work has been put into the fabrication of MMCs with lightweight materials as a matrix in recent years [4].

MMCs are made up of two phases: matrix phase, which is the base material, and the reinforcing phase. The most important component, the matrix, may comprise ceramics, metals, or polymers. The reinforcement can be made of continuous or irregular fibers or particles, and it can be useful to constituents to boost their mechanical, electrical, thermal, wear, and corrosion-resistant properties [5]. Recently, composites have been used in high-temperature industrial materials as well as the automotive, aircraft, electronics, ceramics, biotechnology, and nanotechnology industries [6]. Composites are manufactured using processing techniques adapted to the technological requirements of a given sector. The heat generated by electronics may be decreased by creating special heat sink materials instead of using steel or other alloys. Various constituents have effects on a composite’s properties. Factors include particle size, shape, and dispersion; interactions at the constituent interfaces; constituent phase characteristics; dispersed phase geometry; and particle proportions [7]. The utilization of composite materials in engineering is significantly influenced by their high specific strength and modulus. These characteristics are highly valued in the aerospace and aviation sectors. When structural components comprise composite materials, it results in reduced fuel consumption, increasing the effective characteristics of aircraft [8]. In addition, unlike macroscopic objects of isotropic engineering materials, composite materials allow for the flexible placement of strength and stiffness in crucial areas without compromising weight differences during the fossil fuel crisis [9].

**Figure 1:**
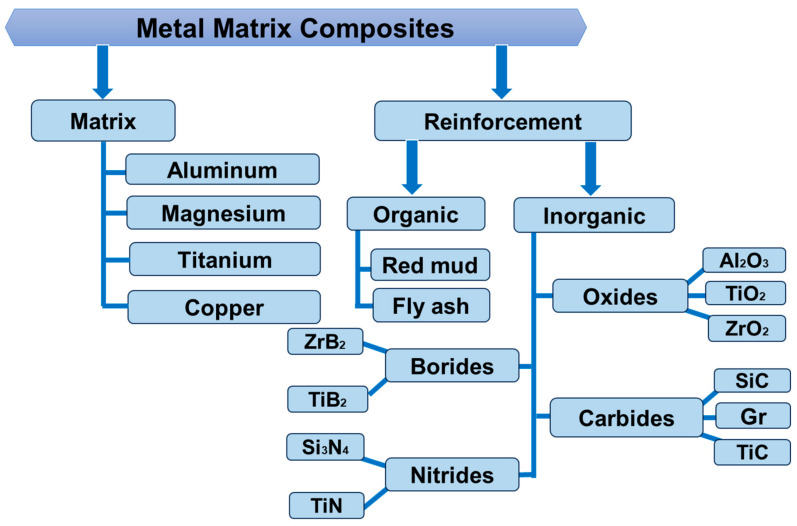
Several kinds of metal matrix composites.

MMCs are usually composed of a distributed ceramic or metallic phase and a metallic matrix. Aluminum (Al), magnesium (Mg), titanium (Ti), etc. are examples of low-density, high-strength-to-weight composite materials that use matrix material reinforced with fibers, whiskers, or particles. Ceramic, metal, or inorganic constituents can be used as reinforcement materials [10]. The various kinds of MMCs with different matrices and reinforcement particles are depicted in Figure 1. The past ten years have seen a rise in interest in MMCs, especially in automotive and general transportation productions owing to their increased explicit stiffness, strength, and reduced susceptibility to galvanic corrosion. Tensile strength and Young’s modulus can be adjusted, as can the corrosion properties by selecting the right composites, which is one benefit of using MMCs as biomaterials [11]. The focus of materials research and development has switched from monolithic to composite materials according to the requirement for structural materials that are high-performing, low-weight, high-quality, and perform well worldwide. Performance, financial, and environmental advantages are what propel MMC use in the automotive and aerospace sectors. Reduced airborne pollution, noise, and fuel consumption are foremost advantages of MMCs in transportation sectors. 

### 1.2. Selection and Fabrication of MMCs

The main constituent of composites is the matrix phase, which is typically composed of a soft metal with a higher tensile strength, shear modulus, and toughness, and lower thermal expansion. Al, Mg, and Ti are the matrices most frequently used in MMCs. Al and Mg are particularly popular because of their special qualities and features, which include a low melting point, good heat conductivity, reasonable mechanical properties, corrosion resistance, lightweight, and cost-efficient manufacturing [12]. A matrix’s primary duties include distributing and shifting the load to the reinforcement. The bonding contact between the matrix and reinforcement determines how much load is transferred, but bonding can also be contingent on the kind of reinforcement and matrix used, as well as the manufacturing process. At present, aluminum alloys are the main focus of the matrix material for MMCs because of their exceptional mechanical properties, low density, and strong corrosion resistance [13].

Reinforcement materials increase the mechanical and thermal characteristics of composites by increasing their tensile strength, compressive strength, rigidity, wear resistance, temperature confrontation, and thermal stability. Particles in the reinforcing phase can be micron- or nanometer-sized and continuous or discontinuous. The aforementioned characteristics of composites, as well as the sustainability of interface bonding and dispersion within the matrix phase, are all related to reinforcement [14]. The required minimal properties of the composite system or the component to be manufactured always dictate the choice of reinforcement. 

**Figure 2:**
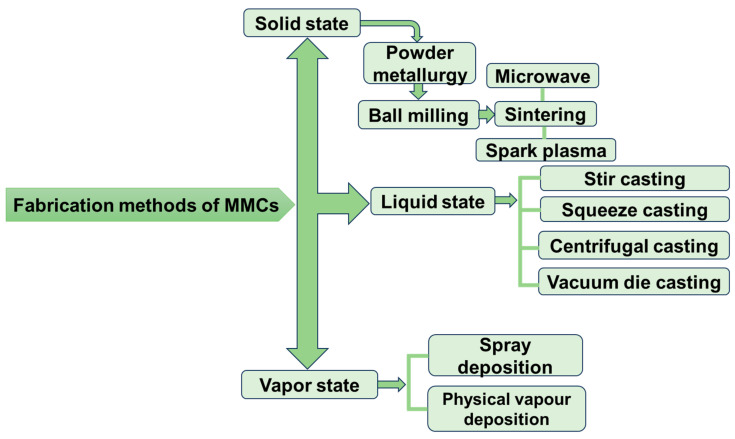
Fabrication methods of metal matrix composites.

Several manufacturing processes have been adopted for the fabrication and synthesis of MMCs. Several methods were employed to improve the results, but the major objective was to deliver a useful, inexpensive, and efficient processing approach where the qualities would be modified. The requirements and characteristics of the finished product place restrictions on the advantages and disadvantages of these procedures. The fundamental techniques fall into three categories: liquid state, solid state, and vapor state methods. Figure 2 displays various fabrication methods of MMCs.

## 2. Application of Metal Matrix Composites

**Figure 3:**
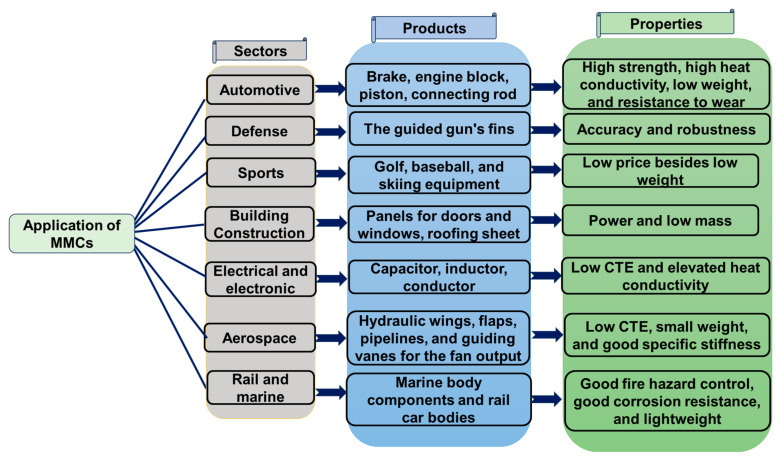
Applications of MMCs in various sectors.

Due to their exceptional qualities, MMCs are finding increased application in the aerospace and automotive sectors. MMCs have better mechanical and physical characteristics to be employed in a variety of automotive parts, including engine blocks, brakes, cylinders, and pistons [15]. Similar to this, MMCs with aluminum matrices in particular are being investigated in the aerospace sector for their potential to improve aircraft structural performance, with the possibility of considerable weight and cost reductions [16]. Due to recent processing advancements that have produced high-quality, reasonably priced components, titanium MMCs are also being investigated for aerospace applications. Ultimately, the extraordinary properties of MMCs like their elevated specific strength, stiffness, and resistance to wear are what drive their usage in a variety of sectors. [17]. Figure 3 presents the applications of MMCs in various sectors with their use in specific products and relevant essential properties [18]. 

Al-based MMCs are significant in automotive and aerospace sectors as they offer better characteristics than base alloys. Aluminum composites’ mechanical and physical qualities are improved by the inclusion of different reinforcing particles, which qualifies them for use in aerospace and automotive applications. In the aerospace and other sectors, MMCs are crucial for the progress of novel structural materials. These composites have outstanding mechanical and physical qualities, greater wear resistance, elevated precise strength, and stiffness. When linked to conventional engineering materials, the metallic matrix’s characteristics are improved by the use of filler reinforcement.

Composite materials, polymers, and ceramics have dominated new materials in numerous sectors over the past ten years. The automotive, agricultural, and mining industries employ MMC components because they need to reduce weight while still achieving good material performance and efficiency. Customers are putting more and more restrictions on fuel efficiency while also expecting higher levels of comfort and safety from the automobile industry. Automotive manufacturers are adopting lighter and more energy-efficient products to fulfill these demands. High-specific stiffness and high-strength MMCs might be employed in long-standing applications where weight decrease is a key component, such as automobile engines, robotics, high-speed rotating shafts, and machinery. 

Over the last five decades, the usage of aluminum in automotive applications has grown by above 80%. It was estimated that by 2015, the entire amount of aluminum vehicles produced in 1996 would increase to 250 or 340 kg, based on whether applications for structure or body parts are taken into account [19]. Strong forecasts exist for aluminum’s use indoors that hangs on a steel frame, trunk lids, and hoods. Engine blocks, which are among the heaviest elements, are being made of aluminum instead of cast iron, which is a major trend that reduces weight significantly. By the year 2000, it was anticipated that 50% or more of all automobiles would have aluminum engine blocks [20]. Automobiles are where aluminum castings are most commonly used. Almost all pistons, around 75% of cylinder heads, 85% of intake manifolds, and the transmission (as well as other components including the rear axle, differential housings, and drive shafts) in automobile power trains are made of aluminum castings [21]. An aluminum alloy is used to make castings for instrument panels, suspension, braking components, brake assemblies, and around 40% of the wheels in chassis applications. Lately, there has been a greater focus on developing wrought aluminum applications as opposed to castings. When more severe loading circumstances and greater mechanical requirements are needed, forged wheels have been employed. Other goods that employ wrought aluminum include heat shields, airbag housings, pneumatic systems, sump pumps, and seat frames. 

Heat exchangers are another common application for aluminum alloys. Over the past 25 years, aluminum’s market share has consistently increased, making it the preferred material for usage in the automobile heat exchanger sector [18]. Many separate heat exchangers are found in modern, high-performance cars, such as those for temperature control, charge air coolers (CACs), and cooling systems for the engine and gearbox. Heat exchangers and castings for engine, transmission, and wheel applications have been the primary uses of aluminum in the automobile sector. The primary barrier to aluminum’s usage in large-scale sheet applications continues to be its cost and price stability. The aluminum sector has allocated substantial resources to assist this endeavor and has set its sights on the automobile sector for future expansion. 

The most potential for weight reduction when employing a substantial amount of aluminum is provided by the body-in-white (BIW). Recent advancements suggest that aluminum might save up to 50% of the weight of steel within the BIW [22]. This can result in a 20–30% drop in the vehicle’s total weight when paired with other weight-saving measures [23].

## 3. Machining Challenges for MMCs

Due to the characteristics associated with matrix and reinforcements, the traditional machining, i.e., turning, drilling, and milling of MMCs, is challenging [24]. Rapid tool wear and high machining costs are the main issues. The most common tool materials used for composite machining include silicon nitride, alumina, cubic boron nitride (CBN), tungsten carbide (WC), polycrystalline diamond (PCD) tools, and chemical vapor depositions (CVDs). Several studies have made it abundantly evident that PCD tools are superior and the only option available to replace MMC machining in terms of improved machinability and longer tool life. When machining MMCs, Weinert and Konig [25] noted that tool wear elevates production costs. Moreover, the main way in which particles traveling over a rake induce wear is by abrasion. It was also concluded that, despite their high cost, CVD-coated diamond tools are superior to PCD tools and are the best option for cutting MMCs in terms of hardness when turning composites, among the several tool materials, such as ceramics, CBN, PCD, and cemented carbide. In terms of performance, only diamond tools were appropriate for this procedure. It was also determined that the work surface roughness increases with an upsurge in feed rate and declines through an upsurge in cutting speed. Even though there is disagreement about precise mechanism underlying tool wear, more study is necessary to enhance the cutting process’ performance and economy. Comparatively speaking to traditional metal machining, very little machining data about composites are available in tool manufacturer catalogs. As a result, adjusting cutting conditions is critical, and researchers have found it to be a time-consuming process thus far. When a cutting tool is used in classical machining, force is applied to remove chips. The tool has a significant setback when cutting hard materials like MMCs, which leads to its failure. 

Komanduri et al. [26] delivered an outline of the development and challenges of machining fiber-reinforced composites, highlighting the need to balance superior properties without compromising weaknesses, and emphasizing the potential of composites to revolutionize industries like the auto industry. Automation is required to save costs and improve competitiveness in the mass production of composite parts since traditional manufacturing procedures are insufficient for the newer composite materials. Pramanik [27] discussed the encounters of traditional machining for MMCs, emphasized the importance of modern methods, and called for further research and optimization in this field. The understanding of certain modern machining methods like electro-discharge, laser beam, and abrasive water jet machining is more advanced and required to be understood and investigated for particulate-reinforced MMCs. 

## 4. Review of Previous Studies on Modern Machining of MMCs

With the development of technology, the field of traditional machining is almost being eliminated. By precisely and perfectly machining difficult-to-machine and intricately structured materials, modern or advanced techniques have demonstrated their viability. Several modern machining processes include laser beam, electrical discharge machining (EDM), electrochemical machining, abrasive water jet machining, ultrasonic machining, and their variants and hybrid processes [28,29]. One of the key factors in determining whether a machining technique can be commercialized is cost. But in exchange for these advantages, there may be few negative environmental and financial implications, such as a low MRR, high specific energy consumption, deteriorated surface properties, dangerous emissions close to the operator’s breathing zone, risk of fire explosion, and production of toxic waste and sludge [30]. The following subsections address some of the prior research on the machining of MMCs employing EDM, laser, abrasive, and hybrid processes. 

### 4.1. Electric Discharge Machining of MMCs

EDM is a modern machining process that is now widely employed by many industries to manufacture molds and dies, aircraft components, automobile parts, and surgical components [31]. Using a tool that introduced an electric release into the workpiece via a dielectric fluid, the workpiece was detached during the development of this process in the early 1940s [32]. The purpose of this controlled procedure was to vaporize the material from the metal surface to machine the component. Using thermal energy of electric sparks, a highly developed noncontact machining technology removes material from the workpiece by melting and vaporizing it. The inclusion of reinforcement material in MMCs slows down and complicates their machining even by EDM-type processes. Because of its competitive machining cost and features, the industry has widely accepted the EDM of MMCs [33]. EDM has significantly advanced the medical, optical, dental, jewelry, and other industries in the past several years. EDM, which erodes a specified quantity of metal by creating millions of electric discharges in a matter of seconds, is one of the more violent but controlled thermal processes among them [34]. Even though there have been many advancements in this field, Abbas et al. [35] found that the goal of the EDM technique is still to increase machining efficiency. It guarantees an acceptable MRR, reduced tool wear, and enhanced surface quality on both a macro and micro scale. These days, there are numerous EDM procedures that are categorized based on the dielectric fluids they employ. Dielectric fluid is a crucial component of the EDM process and affects output and quality significantly. Furthermore, it is not always desirable for the deposited carbon on machined surfaces to diffuse into the workpiece materials and create a solid solution. As an alternative to dielectric fluids based on hydrocarbons, some researchers have experimented with environmentally benign options such as pure water and organic compounds mixed with deionized water [36]. Water offers numerous advantages when used as a dielectric medium in the EDM process. It is non-toxic, eco-friendly, affordable, readily available, and aids in lowering carbon diffusion on machined surfaces. Deionized water has been used as a dielectric in precision machining with promising results, mostly because of its excellent flowability, low viscosity coefficient, and increased thermal conductivity. In addition to producing a surface with fewer flaws and low surface roughness, the use of steam dielectric also lessened the EDM process’s environmental effect and ongoing costs [37]. The use of steam as a dielectric in the EDM of an MMC produced a thinner recast layer. 

**Figure 4:**
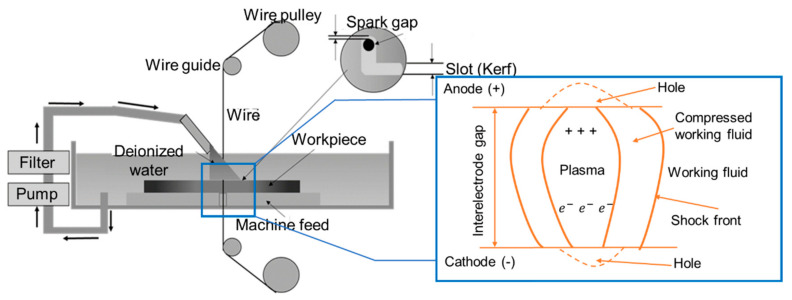
Schematic diagram of WEDM process.

Wire electrical discharge machining (WEDM) is one of the EDM variants that uses a highly conductive metal wire as a tool electrode to accurately cut thick metal components. In essence, WEDM is used to cut conductive metal workpieces with the insignificance of tool wear. Consequently, the tool electrode in WEDM is made of a highly conductive metal wire. Although wires with smaller diameters provide a narrower K_f_, which indicates little material loss, larger-diameter wires have a longer tool life. Figure 4 displays a schematic diagram of the WEDM process. Materials of any shape or hardness can be machined as long as they can conduct electricity. To produce a 2D complicated shape on the workpiece using the WEDM process, only the working table can move in the X and Y directions. However, such WEDM can be used to machine several sophisticated and complicated profiles (like gears, etc.). 

WEDM is capable of precise machining based on the tool path and cutting settings. The spark energy, such as I_p_, and T_on_, has a noteworthy influence. Materials melt and, consequently, evaporate off from the workpiece and tool electrode in high spark intensity [38]. On both materials, the loss of material leaves craters. The generated R_a_ may appear to be a flaw in the formed feature if the generated craters are too coarse for micro-feature manufacturing. Therefore, the spark energy should be in the lowest ranges possible within the machining setup to minimize the unit removal of the material. 

Gore et al. [39] discussed the importance of capacity optimization, costing models, and the machining of MMCs employing WEDM under the concept of ‘Industry 4.0’. Researchers have been attentive to process modeling, process parameters, and materials in the context of WEDM for MMCs. WEDM can effectively cut complex shapes and features out of MMCs. During a machinability study in the EDM/WEDM of MMCs, MRR R_a_ and TWR are crucial output indicators or responses. The economic advantages of utilizing EDM/WEDM expand globally with a higher MRR, lower R_a_, and TWR. 

**Figure 5:**
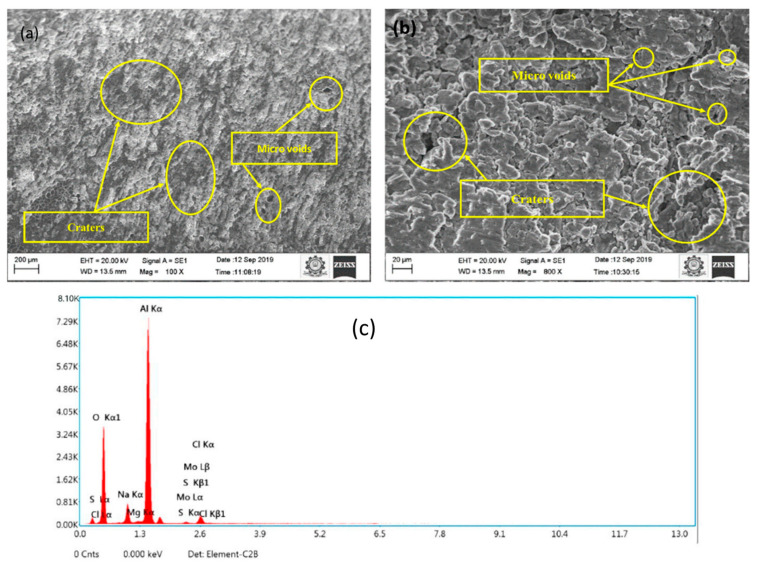
SEM picture at (**a**) 100X (**b**) 800X and (**c**) EDX images of WEDM machined exterior of Al-Mg-MoS_2_ composite in optimum settings [43].

According to Hocheng et al. [40], the material removal rate (MRR) increases initially and then decreases as a result of debris becoming trapped in space amid the tool and the workpiece. A high I_p_ and a shorter T_on_ help to prevent increasing surface roughness, which is associated with a higher MRR when the current increases. Seo et al. [41] studied similar trends using the EDM of SiC-reinforced Al matrix MMCs. According to Patel et al. [42], the size and weight % of SiC-reinforced Al matrix composites were the most important factors to consider while machining these MMCs using micro-EDM. They found that the MRR and EWR increase with servo speed and pulse length but fall when sparking V increases. The machinability characteristics of the Al-Mg-MoS_2_ composites were examined using the WEDM technique [43]. The optimal conditions were I_p_ at 3, T_on_ at 2, and V at 1 for R_a_ and overcut. The SEM and EDX images of WEDM machined surface at optimal environments are shown in Figure 5. Under optimal conditions, there were fewer micro-voids and sinkholes as observed in the SEM images. This was due to increased metal erosion and melting taking place in the machining zone as a result of high spark energy generation brought on by electrostatic and electromagnetic forces at maximum peak current. No discernible reaction is seen in the machined surface’s EDX picture.

Prakash et al. [44] fabricated Al-alloy/Fly ash/B_4_C hybrid composites and considered the machinability using the WEDM process, resulting in V and WF as the most dominant constraints. Al 6063 was reinforced with SiC at 5, 10, and 15% by Satishkumar et al. [12] who observed that with an upsurge in the % of SiC in MMCs, the MRR decreased and R_a_ increased. For achieving a higher MRR, voltage was found to be more vital than the other WEDM process parameters. In the machinability study of Al 2024 MMCs with a SiC of 0.15 and 20% using WEDM, the MRR and R_a_ increased with a rise in T_on_ but decreased with an upsurge in the conformation of SiC [45]. Among all WEDM process parameters, T_on_ was found to be the factor contributing the most, followed by I_p_ and WF. 

The machining of Al 7075 with 3 wt% of TiB_2_ MMCs using the WEDM process was studied by Ahilan and Rajan [46]. When it comes to multi-response optimization, the suggested Grey-based Taguchi approach is helpful. It was revealed that T_on_ (8.5%), WF (14.28%), and T_off_ (74.5%) had the greatest impacts. The extreme MRR of 35.760 mm^3^/min was achieved at T_on_ 1.03 µs, T_off_ 20 µs, I_p_ 200 Amps and R_a_ of 2.015 µm at T_on_ 0.89 µs, T_off_ 20 µs, I_p_ 160 Amps during the machinability study of A413 alloy by the WEDM process [47]. Malhotra et al. [48] compared the performances of different EDM processes in machining hybrid MMCs. Gas-assisted powder mixed EDM (GAPMEDM) showed superior performance for MRR and electrode wear rate (EWR) compared to rotary EDM (REDM) and gas-powered EDM (GAEDM). Alam et al. [49] discussed the successful machining of insulating ceramics using the assisted electrode method (AEM) in the WEDM process, showing effects of different constraints and materials on the machining process. With the inclusion of ethylene glycol and Al_2_O_3_ nanopowder, machining of insulating zirconia ceramic, using the AEM-based WEDM method was effective. Additionally, stainless steel AE produced a larger cut length than copper AE. Vegetable oil was shown to be the ideal dielectric fluid during the machinability study of the Al/Zn/TiC composite utilizing WEDM [50]. Vegetable oil by way of the dielectric fluid showed benefits of optimal machining parameters and the improved surface quality of a WEDM machined surface. 

**Figure 6:**
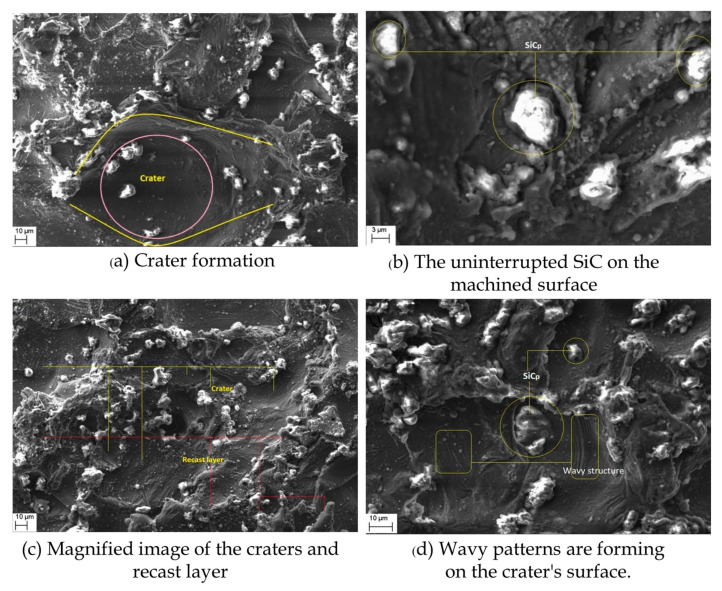
WEDM-induced surface morphology of Al-6061/SiC MMCs [51].

The WEDM machined surfaces of Al 6061/SiC MMCs were morphologically studied using SEM [51]. Figure 6 depicts these craters in magnified perspectives. It appears that the reinforced SiC particles are preserving the integrity of the recast layer because a few of the particles are transferred to the cut surface. The dielectric fluid circulation in the machining process is responsible for this. Rao et al. [52] focused on the impact of machine tools and electrode parameters on EDM performance measures such as MRR and TWR, emphasizing the importance of customized P/M electrodes and the optimization of parameters for enhanced machining efficiency. The suggested strategy reduced the TWR from 7.1 to 5.9 mg/min and increased the MRR from 109.1 to 151.9 mg/min at the optimum combination of parameters. During an investigation of process parameters in WEDM for Al6061/7%SiC/3%B_4_C composites, voltage and T_off_ were the greatest substantial constraints for the MRR and R_a_ [53]. T_on_ is the utmost influential constraint for achieving a higher MRR and lower R_a_ during the WEDM machining of MMCs [54]. The optimal circumstances to attain a higher MRR were determined at a T_on_ of 130 μs, I_p_ of 20 A, and WF of 1 mm/min. The WF of 2 mm/min, I_p_ of 20 A, and T_on_ of 130 μs were found to be the ideal values for attaining an acceptable R_a_. 

Salur et al. [55] discussed the importance of MMCs in industry, the challenges related to their machinability, and the investigation of making constraints on thrust force and R_a_ of MMCs. With a decrease in thrust force and R_a_ values, the feed rate increased during machining. The most optimal parameters for the MRR were found to be an I_p_ of 12 A, T_on_ of 100 µs, and Cu electrodes during EDM machining on TiN-coated Ti6Al4V alloy [56]. The growing discharge current improved the MRR and R_a_ while growing pulse duration and falling discharge current improved TWR. The E-Cu electrode outperformed CuBe electrode in the dispensation of TiN-coated Ti6Al4V. Vishnu et al. [57] discussed the development of regression models for the WEDM of Al-based MMCs, focusing on the MRR and R_a_ as performance measures, using Taguchi’s approach and multiple regression analysis to optimize process parameters for improved performance. T_on_ has the greatest influence constraint for the best process constraints for the MRR and R_a_. The ideal process parameters are T_on_ of 30 µs, T_off_ of 15 µs, and I_p_ of 3 A for better performance. An investigation of machining presentations of Al6061-SiC MMCs employing WEDM was performed, highlighting the effects of process parameters on R_a_ [58]. R_a_ decreases through growth in SiC volume percentages in MMCs, leading to lower average R_a_ values compared to unreinforced Al6061. For unreinforced Al6061, the average R_a_ value was 3.64 µm. The average R_a_ values of Al-6061 MMCs with 5% and 10% SiC were 3.43 and 3.27 µm, respectively. Babu et al. [59] examined the machining features of Al-6061 employing WEDM, identified significant parameters affecting performance measures, determined optimal values for maximizing the MRR, and emphasized the influence of I_p_ on both the MRR and R_a_. The MRR grows with an increase in T_on_ and I_p_, while it decreases during the T_off_. 

The literature indicates that EDM and WEDM have potential to fabricate MMCs and secure improved machinability. The pulse parameters, i.e., current and pulse-on time have been found to be the two most influential parameters that affect surface quality and productivity. A trade-off always exists due to the conflicting responses, i.e., surface quality and productivity, that can be resolved by process optimization.

### 4.2. Laser-Based Machining of MMCs

**Figure 7:**
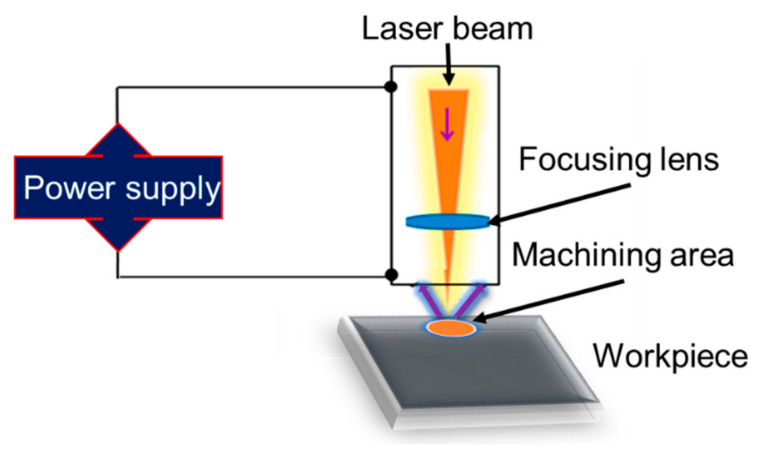
Principles of laser beam machining process.

Recently, there has been a global focus on the creation and manufacture of ceramic components using LASER (light amplification by stimulated emission of radiation)-based manufacturing processes. The basic working principle of the laser beam machining process is illustrated in Figure 7. The efficiency, accuracy, and quality of the material processing are greatly influenced by the laser output properties in laser machining or manufacturing operations [60]. More powerful lasers will make it possible to process MMCs more effectively, which will speed up production and lower manufacturing costs. The creation of small, lightweight laser systems will make it possible to process MMCs on-site or in the field, which will be advantageous for sectors including building, archaeology, and cultural preservation. Water jet-directed laser drilling of Al MMCs removes substances via cold ablation, producing high-quality holes with good circularity and no heat-affected zones or recast layers, making it suitable for industrial applications [61]. Owing to its accuracy, speed, and versatility in handling various materials, laser technology finds extensive use in various industrial processes such as drilling, cutting, welding, and additive manufacturing. Laser machining is capable of achieving dimensional accuracy as high as 0.0005 inches [62]. Kumar et al. [63] discussed the manufacture and study of Al-2024-B_4_C/TiC hybrid MMCs using a laser beam machining process. The addition of reinforcement particles improves mechanical properties but decreases impact strength, while variations in power and time significantly affect hole diameter and roundness during laser machining. This process also produced better-looking machined surfaces. By lessening the pressures of Al_2_O_3_ particles on the cutting tool, the softening effect of laser heating on the Al-based matrix lowers cutting tool wear [64]. By increasing compressive residual stress in the machined surface, laser-assisted hot machining improves surface quality and fatigue strength. Using LBM procedures, the material characteristics of a TiC and WC-reinforced Al MMC were determined. The most important parameters influencing the material deletion rate were found to be the current, WP, and laser power [65].

The presence of SiC elements impacts the machining processes and surface quality. Cold ablation was used to remove the material during water-jet-guided laser drilling of Al-based MMCs, leaving no residual melt layer in the bulk material [61]. The same method of cold ablation is used to remove both soft-matrix and hard particles; this is entirely distinct from the traditional laser drilling method, which ejects both the liquid aluminum and solid SiC without melting at the entry and exit of drilled holes. Padhee et al. [66] heightened the laser drilling process for Al/SiC particulate MMCs by minimizing the taper, spatter, and heat-affected zone (HAZ) simultaneously. They also discussed the challenges in machining MMCs, presented an approach for optimizing Nd: YAG laser drilling of MMCs, and sought to improve particular stiffness and strength for gas turbine engine applications at high temperatures.

### 4.3. Abrasive-Based Machining of MMCs

**Figure 8:**
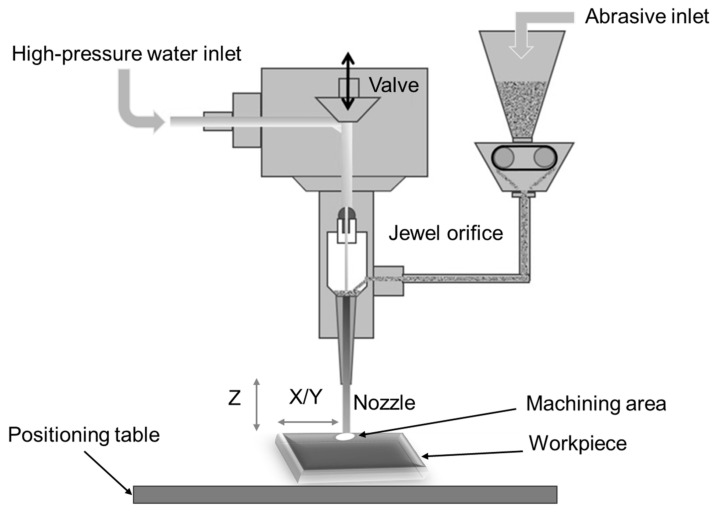
Schematic of machining regions of MMCs during AWJM process.

MMCs can be machined using the abrasive water jet machining (AWJM) technique. The AWJM process is sometimes referred to as environmentally friendly, green machining, or green manufacturing because of its many advantages, which include decreased waste generation, no thermal distortion from the cold cutting mechanism, low cutting forces that do not cause chatter, higher machining flexibility and versatility, less environmental contamination, less sensitivity to changing material properties, and the inability to generate any dust, aerosols, or fumes [67]. A variety of materials, including soft, hard, and reinforced materials, can be machined using an adaptable AWJM method. Some unsolved issues with the AWJM process include poor penetration depth and a low rate of material removal [68]. The MMC machining regions administered to AWJM are depicted in Figure 8. 

**Figure 9:**
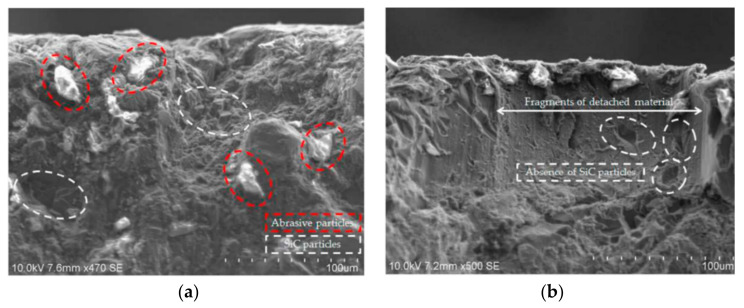
(**a**) Details of material deformation caused by particle impact and (**b**) details of separated material pieces during AWJM process [69].

For the AWJM machining of Al/SiC MMCs, the most advantageous surface quality values with a roughness of around 5 µm were achieved with moderate feed speeds, low to medium pressures, and high abrasive rates [69]. Values in the range of 1° to 2° were found when the taper angle was evaluated. Particles embedded in the material distorted its surface, as seen in Figure 9a. Conversely, Figure 9b demonstrates that material detachments occur in some areas due to the impact of abrasive particles when reinforcing particles are lost. Hardening via plastic deformation is brought about by the constant collision of particles. Shanmugam et al. [70] discussed the importance and benefits of composite materials in various industries and presented an experimental study on cutting procedures for composite constituents. The benefits of composite materials have driven their application growth in diverse sectors. The study demonstrates the potential effectiveness of abrasive water jet, plain water jet, and laser-cutting methods for carbon composite and fiber-reinforced plastic materials, and ductility of the component processes. Sasikumar et al. [71] discussed challenges in machining MMCs and the use of AWJM technology to optimize process parameters and improve kerf characteristics for hybrid Al-7075 composites with 5, 10, and 15% of TiC and B_4_C reinforcement. Traverse speed had the most significant impact on top K_f_, while WP influenced KA and surface finish. Increasing the jet traverse speed decreased top K_f_ and R_a_ but increased KA.

Water jet pressure and traverse speed significantly influence KA and R_a_ in the AWJM of hybrid MMCs. Interactions between hard ceramic (B_4_C) and soft lubricant (hBN) particles affect the cutting behavior of the composites [72]. Traverse speed is the most influential parameter on the MRR and WP on R_a_ while machining Al/SiC MMCs using AWJM [73]. The optimum condition in the AWJM process was found to be WP of 2800 Bar, AF of 400 mg/min, FR of 1000 mm/min, and SOD of 4 mm for all the response measures. The optimization of the AWJM process parameters using the Taguchi-based ANOVA method to reduce R_a_ and the focus on Al-based MMCs and their machining challenges were performed by Maneiah et al. [74]. The feed rate and abrasive flow rate were identified as the most significant parameters affecting R_a_. R_a_ was also greatly impacted by the SOD. The ideal process parameters to obtain a low R_a_ on produced composites were 135 mm/min of FR, 0.5 SOD, and 450 gm/min of AFR.

Higher CNT concentrations in aluminum alloy composites improve mechanical properties significantly, affect machining parameters like the MRR, R_a,_ and KA, and require specific machining conditions for optimal performance. Other parameters had equal contributions to the MRR and KA, but SOD had a significant effect on R_a_. The ideal parameters for machining Al-based MMCs with CNT are a stand-off distance of 1 mm, WP of 350 N/mm^2^, and speed of 30 mm/min [75]. While machining Al 6063-based MMCs with (5%, 10%, and 15%) volume fraction of B_4_C employing the AWJM process, combinations of 80 mesh size, 340 g/min of mass flow rate, 200 MPa of WP, and 60 mm/min of traverse speed resulted in a higher depth of cut [76]. Hamatani and Ramulu [77] presented an investigational study on the machinability of ceramic and MMCs using an abrasive water jet, concluding that Al/SiC composite is easily machinable, TiB_2_/SiC ceramic composite is feasible for abrasive waterjet machining, and slotted edge damage in MMC is larger than in ceramic composites.

There is an increasing trend of hybrid machining processes that can significantly increase productivity, improve surface layer properties, reduce tool wear, and decrease energy consumption [78,79]. A few noteworthy research emphasized the significance of the LBM-EDM hybrid process as well as the improvement of vibrations and the magnetic field for better MMC machinability [79,80,81]. Based on the properties of MMCs and characteristics of the machining processes as well as on the desirable outputs, the machining of various MMCs has been attempted. Table 1 presents various MMC types machined using three different processes considered in the present work. 

## 5. Conclusions and Future Research Directions

This paper has presented a detailed review of recent developments in modern machining of MMCs. It is evident that the unique characteristics of MMCs such as exceptional strength-to-weight ratio, thermal conductivity, dimensional stability, low specific density, low coefficient of thermal expansion, and high specific stiffness make them superior material choices for applications in the railroad, aerospace, automotive, bio-medical, and marine sectors. Various technological challenges in the machining of MMCs are being addressed by research, innovations, and developments in modern machining processes. It can be summarized that to achieve precision machining and retain requisite mechanical integrity, further technological interventions are needed where researchers must find new ways to machine MMCs efficiently without sacrificing the intrinsic qualities of the MMCs composite.

The following are important conclusion points:

⮚ Among the several machining techniques, EDM has become the most common due to the improvements it has brought about in recent years. MMC performance during machining is greatly impacted by its composition. Al_2_O_3_, Gr, and SiC are a few examples of reinforcing materials that have demonstrated varying impacts on machining parameters like the MRR and R_a_. Based on the intended machining results, great consideration must be given to the selection of reinforcing type and content.
⮚ To increase surface quality and machining efficiency, parameters of the machining process must be optimized. Researchers have examined the intricate interactions between process parameters, highlighting the need for MMCs to use specific settings to avoid material deterioration.
⮚ Excessive heating during thermal machining like EDM and laser beam machining tends to melt and evaporate the reinforced particles and matrix material. In some cases, the thermal damage and formation of a recast layer are unavoidable. In a fiber-reinforced composite, the plastic deformation of the matrix material causes fiber pullout and fracture. By employing strategies like dry EDM, process optimization, effective flushing techniques, etc., these issues can be addressed.
⮚ The use of water as a dielectric instead of oil has been introduced in EDM because of concerns about the environment, human health, and safety during the machining process. More studies are required on various EDM interventions for the machinability enhancement of MMCs.
⮚ The use of a magnetic field, abrasives, and vibration assistance in conjunction with other hybrid manifestations can be a prolific and effective strategy that can strengthen the capabilities of these processes. To make modern machining technology more competitive, additional research and development are needed for cost, quality, and environmental benefits.

## Figures and Tables

**Table 1:** Various MMCs machined by modern machining processes.

Modern Machining Process	Machined MMCs
Micro-EDM	Al-SiC
WEDM	Al-Mg-MoS_2,_ Al-alloy/Fly ash/B_4_C, Al6063/SiC, Al2024/SiC, Al7075/TiB_2,_ Al/Zn/TiC, Al 6061/SiC, Al6061/7%SiC/3%B_4_C,
Laser-Based Machining	Al-2024-B_4_C/TiC, Al/SiC, Al/WC/TiC
Abrasive-Based Machining	Al/SiC, Al7075/TiC/B_4_C, Al/CNT, Al6063/B_4_C, Al/TiB_2_/SiC

## Data Availability

The research data will be made available upon request.

## Nomenclature

Peak current	*I_p_*	Wire feed	*WF*
Pulse on time	*T_on_*	Abrasive flow rate	*AF*
Pulse off time	*T_off_*	Water pressure	*WP*
Material removal rate	*MRR*	Gap voltage	*V*
Surface roughness	*R_a_*	Stand-off distance	*SOD*
Kerf width	*K_f_*	Kerf angle	*KA*
Tool wear rate	*TWR*	Flow rate	*FR*
Electrode wear rate	*EWR*		
